# Tuberculosis control in China: use of modelling to develop targets and policies

**DOI:** 10.2471/BLT.15.154492

**Published:** 2015-09-15

**Authors:** Hsien-Ho Lin, Lixia Wang, Hui Zhang, Yunzhou Ruan, Daniel P Chin, Christopher Dye

**Affiliations:** a Institute of Epidemiology and Preventive Medicine, College of Public Health, National Taiwan University, Rm706, No.17 Xuzhou Rd, Taipei 100, Taiwan, China.; b Chinese Center for Disease Control and Prevention, Beijing, China.; c China Office, Bill & Melinda Gates Foundation, Beijing, China.; d Office of the Director General, World Health Organization, Geneva, Switzerland.

## Abstract

It is unclear if current programmes in China can achieve the post-2015 global targets for tuberculosis – 50% reduction in incidence and a 75% reduction in mortality by 2025. Chinese policy-makers need to maintain the recent decline in the prevalence of tuberculosis, while revising control policies to cope with an epidemic of drug-resistant tuberculosis and the effects of ongoing health reform. Health reforms are expected to shift patients from tuberculosis dispensaries to designated hospitals. We developed a mathematical model of tuberculosis control in China to help set appropriate targets and prioritize interventions that might be implemented in the next 10 years. This model indicates that, even under the most optimistic scenario – improved treatment in tuberculosis dispensaries, introduction of a new effective regimen for the treatment of drug-susceptible tuberculosis and optimal care of cases of multidrug-resistant tuberculosis – the current global targets for tuberculosis are unlikely to be reached. However, reductions in the incidence of multidrug-resistant tuberculosis should be feasible. We conclude that a shift of patients from tuberculosis dispensaries to designated hospitals is likely to hamper efforts at tuberculosis control if cure rates in the designated hospitals cannot be maintained at a high level. Our results can inform the planning of tuberculosis control in China.

## Résumé

Il est difficile de savoir si les programmes actuellement menés en Chine permettront d'atteindre les objectifs mondiaux pour l'après-2015 concernant la tuberculose, qui consistent à réduire l'incidence de 50% et la mortalité de 75% d'ici à 2025. Les dirigeants chinois doivent confirmer le récent déclin de la prévalence de la tuberculose, mais aussi revoir les politiques de lutte pour faire face à une épidémie de tuberculose pharmacorésistante et les effets de l'actuelle réforme de la santé. La réforme de la santé est censée prévoir le transfert des patients traités dans des dispensaires antituberculeux vers des hôpitaux expressément désignés. Nous avons élaboré un modèle mathématique de lutte contre la tuberculose en Chine qui aide à définir les objectifs appropriés et à hiérarchiser les interventions qui pourraient être réalisées au cours des dix prochaines années. Ce modèle indique que même dans le scénario le plus optimiste – amélioration du traitement dans les dispensaires antituberculeux, introduction d'un nouveau schéma thérapeutique efficace pour le traitement de la tuberculose sensible et traitement optimal des cas de tuberculose multirésistante –, il paraît difficile d'atteindre les objectifs mondiaux actuels pour la tuberculose. Néanmoins, il devrait être possible de réduire l'incidence de la tuberculose multirésistante. Nous en concluons que le transfert des patients traités dans des dispensaires antituberculeux vers des hôpitaux expressément désignés est susceptible d'entraver les efforts de lutte contre la tuberculose s'il est impossible de maintenir des taux de guérison élevés dans ces hôpitaux. Nos résultats peuvent servir de base à la planification de la lutte contre la tuberculose en Chine.

## Resumen

No está claro si los programas actuales en China pueden alcanzar los objetivos globales para la tuberculosis después de 2015, una reducción del 50% de la incidencia y una reducción del 75% de la mortalidad de aquí a 2025. Los responsables políticos de China necesitan mantener el reciente descenso en la prevalencia de la tuberculosis, al mismo tiempo que revisan las políticas de control para hacer frente a una epidemia de tuberculosis farmacorresistente y los efectos en la reforma sanitaria en curso. Se espera que las reformas sanitarias trasladen los pacientes de los dispensarios para tuberculosis a los hospitales designados. Se ha desarrollado un modelo matemático de control de la tuberculosis en China para ayudar a establecer los objetivos apropiados y priorizar las intervenciones que podrían implementarse en los próximos diez años. Este modelo indica que, incluso en el escenario más optimista (una mejora del tratamiento en los dispensarios para tuberculosis, la introducción de un nuevo y efectivo régimen para el tratamiento de la tuberculosis farmacosensible y la atención óptima en casos de la tuberculosis farmacorresistente), es muy poco probable que se cumplan los objetivos actuales globales para la tuberculosis. Sin embargo, las reducciones en la incidencia de la tuberculosis farmacorresistente deberían ser factibles. Se concluye que es posible que un cambio de los pacientes de los dispensarios para tuberculosis a los hospitales designados obstaculice los esfuerzos de un control para la tuberculosis si las tasas de cura en los hospitales designados no pueden mantenerse en un nivel alto. Nuestros resultados pueden dar información sobre la planificación del control de la tuberculosis en China.

## ملخص

من غير الواضح ما إذا ما كان تحقيق الأهداف العالمية المنشودة لما بعد 2015 ممكنًا فيما يتعلق بمرض السل في الصين من خلال البرامج الحالية، حيث تتمثل هذه الأهداف في تقليل حالات الإصابة بالمرض بنسبة 50% وتقليل حالات الوفاة الناتجة عنه بنسبة 75% مع حلول عام 2025. وإن صناع القرار الصينيين بحاجة إلى الحفاظ على مستوى انخفاض انتشار مرض السل، في الوقت الذي يراجعون فيه سياسات السيطرة على المرض للتعامل بفعالية مع وباء مرض السل المقاوم للأدوية وآثار الإصلاح المستمر في مجال الخدمات الصحية، ومن المتوقع أن تؤدي الإصلاحات في الخدمات الصحية إلى نقل المرضى من مستوصفات علاج السل إلى مستشفيات محددة. وقد وضعنا نموذجًا حسابيًا للسيطرة على مرض السل في الصين للمساعدة في تحديد الأهداف المناسبة وترتيب أولويات التدخلات التي يمكن تنفيذها على مدى العشر سنوات القادمة. ويشير هذا النموذج إلى أنه حتى في حالة حدوث أكثر السيناريوهات تفاؤلًا – ألا وهي تحسين سبل العلاج في مستوصفات علاج مرض السل، وتوفير نظم جديدة فعالة لعلاج السل المستجيب للأدوية، وتوفير الرعاية الصحية المثلى لحالات الإصابة بالسل المقاوم للأدوية المتعددة – من المستبعد الوصول إلى الأهداف العالمية الحالية المتعلقة بمرض السل. وعلى الرغم من ذلك، يجب أن يكون الحد من حالات الإصابة بمرض السل المقاوم للأدوية المتعددة ممكنًا. ونخلص من ذلك بأن نقل المرضى من مستوصفات علاج السل إلى مستشفيات محددة من المرجح أن يعيق الجهود الرامية إلى السيطرة على مرض السل في حالة تعذر الحفاظ على ارتفاع مستوى معدلات التعافي في المستشفيات المحددة. ويمكن الاستفادة من النتائج التي توصلنا إليها في توفير المعلومات اللازمة للتخطيط للسيطرة على مرض السل في الصين.

## 摘要

我们尚不清楚中国目前的方案是否可以实现 2015 年后的全球结核病目标——到 2025 年前将发病率降低 50％ 并将死亡率降低 75％。中国的政策制定者需要保持结核病患病率近期的下降幅度，同时修订调控政策以应对耐药结核病的广泛传播以及对持续进行的医疗改革的影响。 医疗改革有望将患者从结核病防治所转移到指定医院。 我们开发了中国控制结核病的数学模型，以帮助设定适当的目标和优先考虑干预措施，这些都可能会在未来 10 年内实现。 此模型表明，即使在最乐观的情况下——改善结核病防治所中的治疗，引进能够有效治疗药物敏感结核病的新方案和耐多药结核病病例的最佳护理——仍不可能实现目前的全球结核病目标。 然而，降低耐多药结核病的发病率应该是可行的。 我们得出的结论是，如果指定医院中治愈率不能保持在较高的水平，将患者从结核病防治所转移到指定医院有可能会妨碍结核病控制工作。 我们的研究结果可以告知中国的结核病防治规划。

## Резюме

Остается неясным, сумеют ли программы, действующие сейчас в Китае, обеспечить достижение глобальных целей в отношении туберкулеза, намеченных на 2015 год, а именно достижение 50%-го сокращения частоты возникновения заболевания и 75%-го снижения смертности к 2025 году. Лица, ответственные в Китае за принятие нормативных решений в стране, должны поддерживать достигнутое за последний период снижение частоты возникновения туберкулеза, при этом им необходимо пересмотреть принципы контроля над заболеванием, чтобы иметь возможность справиться с эпидемией лекарственно-устойчивого туберкулеза в условиях одновременно осуществляемой реформы здравоохранения. Ожидается, что в ходе реформы пациенты с туберкулезом будут переведены из диспансерных заведений в специализированные больницы. Нами была разработана математическая модель контроля над туберкулезом в Китае, которая помогает установить соответствующие цели и установить приоритеты на виды медицинских вмешательств, которые следует осуществить на протяжении 10 лет. Модели указывают на то, что даже в самом оптимистическом сценарии, то есть при улучшении лечения в туберкулезных диспансерах, введении новой эффективной схемы лечения туберкулеза, реагирующего на лекарственное лечение, и при оптимальном уходе за больными туберкулезом со множественной лекарственной устойчивостью, маловероятно, что текущие глобальные цели в отношении этого заболевания будут достигнуты. Однако остается возможность уменьшения частоты случаев заболевания туберкулезом со множественной лекарственной устойчивостью. По нашему мнению, перевод пациентов из туберкулезных диспансеров в специализированные больницы, вероятно, затруднит усилия по контролю над туберкулезом, если только показатели излечения в этих больницах не будут поддерживаться на высоком уровне. Данные результаты исследования могут быть использованы при планировании контроля над туберкулезом в Китае.

## Introduction

In China, between 1990 and 2010, the prevalence of smear-positive tuberculosis and tuberculosis-related mortality fell by 63% and 80%, respectively – one of the most rapid declines in tuberculosis morbidity and mortality in the world.^1^ These improvements have primarily been attributed to the nationwide scale-up of a tuberculosis control programme using short-courses of directly observed treatment, implemented by the Chinese Center for Disease Control and Prevention (CCDC).^1^Between 2000 and 2010, this scale-up was associated with a substantial increase in the proportion of people treated by the Chinese CDC and this increase appears to have played a critical role in reducing tuberculosis prevalence.

People with tuberculosis can access care either via the Chinese CDC system of tuberculosis dispensaries or the hospital system. The Chinese CDC system generally diagnoses and treats patients according to national guidelines on tuberculosis control. The hospital system’s employees are supposed to report and refer tuberculosis to the Chinese CDC but some people are still treated in the hospital system without strict adherence to the national guidelines. Whereas people treated by the Chinese CDC have high rates of treatment completion and success, people treated by hospitals often do not take their medications regularly or discontinue treatment prematurely.^2^

Despite the progress it has made in tuberculosis control, China is facing a serious epidemic of drug-resistant tuberculosis. A national survey of drug-resistant tuberculosis indicated that, in 2007, there were about 110 000 new diagnoses of multidrug-resistant (MDR) tuberculosis (5.7% of total diagnoses), while the prevalence of MDR-tuberculosis among those people who had previously been treated for tuberculosis was 25.6%.^3^ Although MDR tuberculosis can develop as the result of unsuccessful treatment in the hospital system, a large proportion of all incident MDR tuberculosis probably results from person-to-person transmission. An effective programme to prevent as well as to diagnose and treat MDR tuberculosis is urgently needed in China.

The World Health Assembly recently agreed upon new post-2015 global targets for tuberculosis control: global reductions in tuberculosis incidence and mortality by 50% and 75%, respectively, between 2015 and 2025.^4^ It is unclear if China can achieve these new targets, especially given the growing epidemic of MDR tuberculosis. Fortunately, innovations in tuberculosis diagnosis and treatment are either already available or will soon be available. One major concern is that, as part of ongoing health reform, the government is gradually shifting tuberculosis treatment from the Chinese CDC system to designated hospitals. This shift may adversely affect tuberculosis control if the designated hospitals cannot match or exceed the quality of care currently provided by the Chinese CDC.^5^

To help policy-makers set appropriate tuberculosis-control targets and prioritize the related interventions that might be implemented in the next 10 years, we model the potential impact of current and alternative control measures on the epidemiology of tuberculosis in China. In this paper we describe this analysis and its potential impact on future policies and interventions for tuberculosis control in China.

## Model-based analysis

The analysis was developed by a group of tuberculosis modellers, officers of the Chinese CDC and Chinese experts on tuberculosis control. The engagement of policy and field experts ensured that key policy questions were addressed and country-specific contexts – e.g. the different health systems available for tuberculosis care – were reflected in the analysis. Following the suggestions of the policy and field experts, five main scenarios for the future control of tuberculosis were analysed. These scenarios represent the range of policies and interventions that are being considered and could be implemented in China over the next 10 years (Box 1).

Box 1 **Scenarios of tuberculosis control considered in the modelled analysis, China, 2015**

**Scenario 1: Status quo**

The current control programme remains unchanged. Most people (80%) are treated in the Chinese Center for Disease Control and Prevention (CDC) system with the rest treated in the hospital system.^a^ There is no detection or treatment of multidrug-resistant (MDR) tuberculosis.^a^ The long-term cure rates for new treatment and retreatment are 82% and 75%, respectively, in the Chinese CDC system and 55% and 55%, respectively, in the hospital system.^a^ The long-term cure rates for MDR tuberculosis using first-line drugs are 35% in the Chinese CDC system and 30% in the hospital system.^a^

**Scenario 2: Patient shift from Chinese CDC to designated hospitals**

The 80% of people currently treated in the Chinese CDC system are shifted to designated hospitals while the 20% currently treated in the hospital system are treated in public hospitals other than the designated ones. The designated hospitals have lower cure rates than the Chinese CDC system but higher cure rates than the other public hospitals: 70% for new treatment and 65% for retreatment.^a^ The long-term cure rate for MDR tuberculosis using first-line drugs is 30% in all of the public hospitals.

**Scenario 3A: Improving treatment outcome for drug-susceptible (DS) tuberculosis by referral to Chinese CDC system**

People currently treated in the hospital system are referred to – and treated in – the Chinese CDC system. Every person with tuberculosis is therefore treated in the Chinese CDC system.

**Scenario 3B: Improving treatment outcome for DS-tuberculosis by use of new treatment regimen**

The treatment outcomes for DS-tuberculosis, in both the Chinese CDC and hospital systems, are improved by the use of a new and better treatment regimen and the use of adherence technologies.^6^^–^^8^ The long-term cure rates in both systems become 92% for new treatment and 90% for retreatment.^a^

**Scenario 4A: Use of the currently best programmes for the diagnosis and treatment of MDR-tuberculosis**

Most smear-positive patients (90%) received rapid molecular testing for drug resistance and 70% of patients detected with MDR tuberculosis are placed on second-line therapy. The long-term cure rate for MDR tuberculosis with second-line therapy is 60%.^a^

**Scenario 4B: Use of optimized programmes for the diagnosis and treatment of MDR tuberculosis**

New diagnostic tools – e.g. rapid molecular testing – and new treatment regimen for MDR tuberculosis are widely and effectively applied. Most tuberculosis patients (90%) receive resistance testing, 85% of people with MDR tuberculosis are placed on a new and more effective treatment regimen, and the long-term cure rate for MDR-tuberculosis on the new regimen is 82%.^6^^,^^9^^–^^11^

**Scenario 5: Combination of Scenarios 3A, 3B and 4B**

Every person with tuberculosis is treated in the Chinese CDC system, the treatment outcomes for DS-tuberculosis are improved by the use of a new and better treatment regimen and adherence technologies, and the detection and treatment of MDR tuberculosis is improved.

^a^ The parameters are based on the unpublished opinions or estimates of a group of Chinese experts on tuberculosis.

In scenario 1 – the status quo scenario – 80% of tuberculosis patients receive treatment in the Chinese CDC system and the other 20% are treated in the hospital system. In scenario 2, the delivery of tuberculosis services is shifted from the Chinese CDC to public hospitals that have been designated as tuberculosis service providers.^5^ The long-term cure rate for tuberculosis in these designated hospitals was set lower than that in the Chinese CDC system. In scenario 3, the treatment outcome for drug-susceptible tuberculosis – defined as those without the MDR disease – is improved when either, in scenario 3A, 100% of tuberculosis patients are treated in the Chinese CDC system or, in scenario 3B, a new drug regimen along with new technologies to improve treatment adherence are used.^6^^–^^8^ A new diagnosis and treatment programme for MDR tuberculosis is implemented in both scenarios 4A and 4B.^6^^,^^9^^–^^11^ Scenario 5 represented a potentially optimal situation in which scenarios 3A, 3B and 4B are delivered simultaneously. In all of our analyses we ignored active detection of people with tuberculosis and prophylactic treatment for latent infections. All the interventions were assumed to be implemented from 2015.

### Model of transmission

**Fig. 1:**
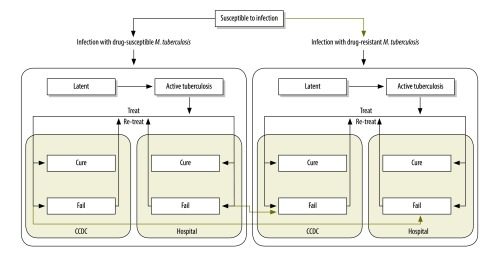
The structure of the dynamic compartmental model of tuberculosis in China

We used a dynamic compartmental model of tuberculosis transmission^12^^–^^14^ in which the whole population is divided into mutually exclusive compartments based on the natural history of tuberculosis – i.e. each individual is deemed susceptible, to have latent infection or active disease, to have been cured after treatment, to have failed or defaulted on treatment, or relapsed after treatment (available from the corresponding author). We categorized the treatment of active tuberculosis as in the Chinese CDC system or in the hospital system. The model was further stratified by two different phenotypes of tuberculosis: drug-susceptible and MDR (Fig. 1). Some of the values we used for the input parameters of the model – e.g. the rate of progression from latent infection to active disease – were based on the results of epidemiological studies of tuberculosis but other values – e.g. the long-term cure rates of drug-susceptible tuberculosis and MDR tuberculosis in the Chinese CDC and hospital systems – were based on the consensus of the national experts (Box 1). The model was calibrated to the reported tuberculosis epidemic in China, using the Bayesian melding approach, accounting for uncertainty in the input parameters.^15^^–^^17^

CCDC: Chinese Center for Disease Control and Prevention.

Note: The green lines represent pathways in which drug-resistant tuberculosis can be generated.

Using the calibrated model, we projected the impact of alternative control interventions on the epidemiology of tuberculosis. The cumulative reduction of tuberculosis incidence and mortality between 2015 and 2025 was projected for each scenario and compared with the post-2015 targets for global tuberculosis control.^4^ For each scenario, we also projected the absolute prevalence of MDR tuberculosis in the general population and among all people with tuberculosis.

Using tornado diagrams and univariable uncertainty analyses, we explored the influence of uncertainty in our parameter values on the reduction of tuberculosis and MDR tuberculosis. Using the posterior resamples from the Bayesian melding procedure, we also conducted multivariable uncertainty analysis of the additional reduction in tuberculosis and MDR tuberculosis under each scenario. We determined 95% credible intervals (CrI) from the posterior simulations. Details of the uncertainty analysis are available from the corresponding author.

## Projected impact

### Scenario 1

**Fig. 2:**
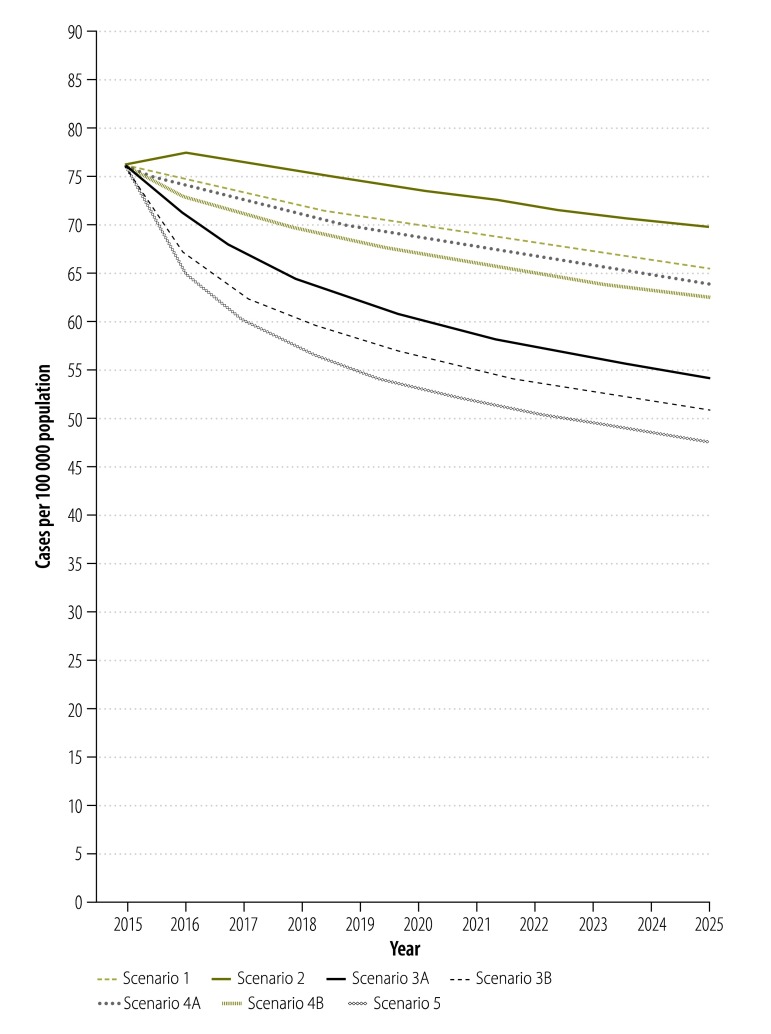
Projected impact of different scenarios of tuberculosis control on the prevalence of pulmonary tuberculosis, China, 2015–2025

Note: The scenarios are as follows: 1: status quo; 2: shift from CCDC to hospital; 3A: all treated in CCDC; 3B: improved treatment of DS-TB; 4A: current best MDR-TB program; 4B: optimized MDR-TB program; 5: combined intervention.

**Fig. 3:**
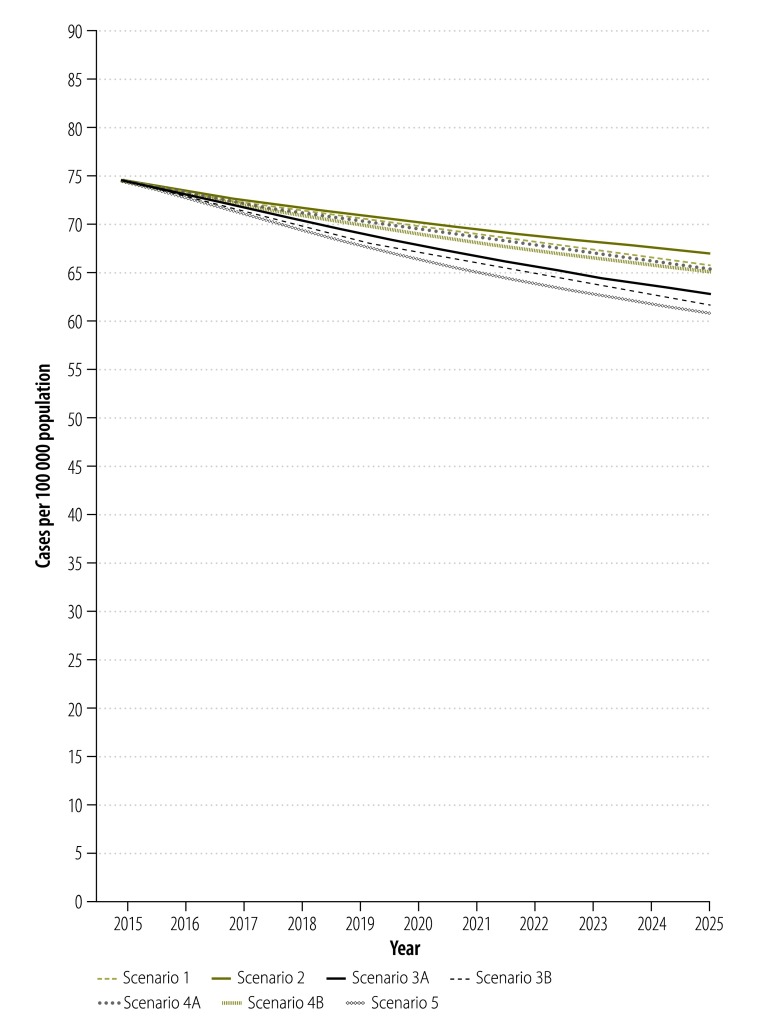
Projected impact of different scenarios of tuberculosis control on the annual incidence of pulmonary tuberculosis, China, 2015–2025

Note: The scenarios are as follows: 1: status quo; 2: shift from CCDC to hospital; 3A: all treated in CCDC; 3B: improved treatment of DS-TB; 4A: current best MDR-TB program; 4B: optimized MDR-TB program; 5: combined intervention.

**Fig. 4:**
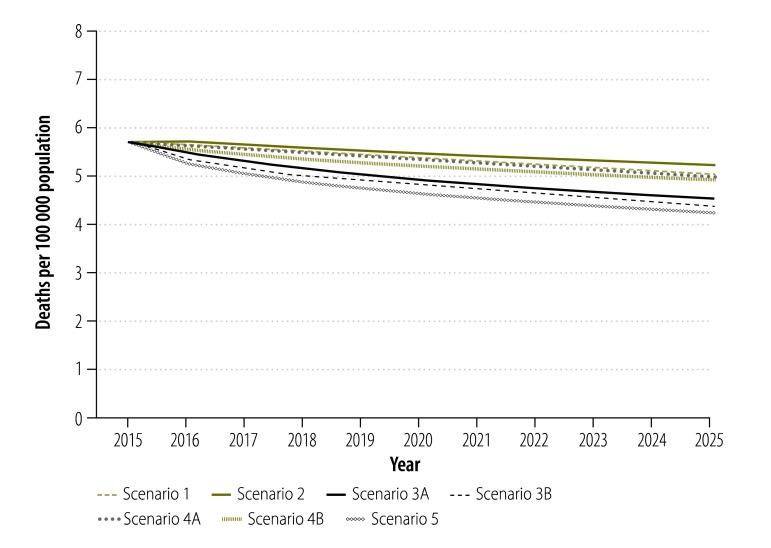
Projected impact of different scenarios of tuberculosis control on mortality associated with pulmonary tuberculosis, China, 2015–2025

Note: The scenarios are as follows: 1: status quo; 2: shift from CCDC to hospital; 3A: all treated in CCDC; 3B: improved treatment of DS-TB; 4A: current best MDR-TB program; 4B: optimized MDR-TB program; 5: combined intervention.

For scenario 1, the dynamic model captured the observed declining trend in tuberculosis incidence, prevalence and mortality as well as the epidemic of MDR tuberculosis. The overall prevalence of tuberculosis (Fig. 2) was projected to continue its current downward trend because effective surveillance and treatment reduces transmission and brings the basic reproductive rate (*R*_0_) below 1. Between 2015 and 2025, however, tuberculosis incidence and mortality in China (Fig. 3 and Fig. 4) were projected to fall by only 11.6% (95% CrI: 9.4 to 14.1%) and 13.0% (95% CrI: 10.4 to 15.6%), respectively (Table 1).

CCDC: Chinese Center for Disease Control and Prevention; DS-TB: drug-susceptible tuberculosis; MDR-TB: multidrug-resistant tuberculosis.

CCDC: Chinese Center for Disease Control and Prevention; DS-TB: drug-susceptible tuberculosis; MDR-TB: multidrug-resistant tuberculosis.

CCDC: Chinese Center for Disease Control and Prevention; DS-TB: drug-susceptible tuberculosis; MDR-TB: multidrug-resistant tuberculosis.

**Table 1:** Projected impacts of different intervention scenarios on tuberculosis epidemiology in China between 2015 and 2025

Scenario	Incident tuberculosis				Tuberculosis-related death		
	Cumulative No., in thousands (95% CrI)	Cumulative reduction, % (CrI)	No. prevented, in thousands (95% CrI)^a^		Cumulative No., in thousands (95% CrI)	Cumulative reduction, % (CrI)	No. prevented, in thousands (95% CrI)^a^
1	9 949 (8 277 to 11 371)	11.6 (9.4 to 14.1)	0 (0)		765 (513 to 964)	13.0 (10.4 to 15.6)	0 (0)
2	10 057 (8 356 to 11 491)	10.0 (6.9 to 12.5)	−107 (−197 to −52)^b^		790 (525 to 981)	9.4 (4.6 to 13.0)	−25 (−44 to −12)^b^
3A	9 712 (8 062 to 11 136)	15.6 (12.9 to 19.6)	237 (177 to 333)		708 (471 to 912)	22.3 (18.4 to 27.9)	58 (34 to 85)
3B	9 599 (7 953 to 10 930)	17.0 (14.1 to 20.7)	351 (261 to 529)		684 (451 to 897)	25.1 (20.5 to 32.0)	82 (58 to 126)
4A	9 914 (8 248 to 11 336)	12.1 (9.9 to 15.0)	36 (16 to 77)		757 (508 to 958)	14.2 (11.7 to 17.1)	8 (4 to 17)
4B	9 884 (8 224 to 11 304)	12.6 (10.3 to 15.9)	66 (31 to 148)		749 (501 to 952)	15.2 (12.6 to 18.7)	16 (9 to 31)
5	9 515 (7 883 to 10 852)	18.2 (15.5 to 22.5)	434 (317 to 634)		664 (437 to 881)	27.9 (22.2 to 36.0)	101 (71 to 153)

CrI: credible interval.

^a^ Compared with the status quo of scenario 1.

^b^ Negative values indicate an increase in the tuberculosis burden.

### Scenarios 2 to 5

In scenario 2, shifting tuberculosis treatment from the Chinese CDC system to designated hospitals had a negative impact on the tuberculosis epidemic, since we defined the cure rate of patients in the designated hospitals as lower than that in the Chinese CDC system (Fig. 2, Fig. 3 and Fig. 4). Under this scenario, the cumulative reduction of tuberculosis incidence and mortality by 2025 would only be 10.0% (95% CrI: 6.9 to 12.5%) and 9.4% (95% CrI: 4.6 to 13.0%), respectively (Table 1). Compared with scenario 1, scenario 2 would result in 107 000 people with tuberculosis and 25 000 additional tuberculosis-related deaths in the decade beginning in 2015 (Table 1).

Further improvement in the treatment outcomes for drug-susceptible tuberculosis would accelerate the decline in the general tuberculosis epidemic (Fig. 2, Fig. 3 and Fig. 4). If, as in scenario 3A, all patients in the hospital system were shifted to the Chinese CDC system, the reductions in tuberculosis incidence and mortality would be improved to 15.6% (95% CrI: 12.9 to 19.6%) and 22.3% (95% CrI: 18.4 to 27.9%;), respectively, by 2025 (Table 1). If, as in scenario 3B, the long-term cure rate could be raised to over 90%, through the use of new tuberculosis treatments and adherence technologies – then tuberculosis incidence and mortality would be expected to fall by 17.0% (95% CrI: 14.1 to 20.7%) and 25.1% (95% CrI: 20.5 to 32.0%), respectively, by 2025 (Table 1). Compared with scenario 1, such improvement in the treatment of drug-susceptible tuberculosis could prevent 237 000 to 351 000 new people with tuberculosis and 58 000 to 82 000 deaths from tuberculosis in the decade beginning in 2015 (Table 1).

As seen in scenarios 4A and 4B, provision of diagnosis and treatment for MDR tuberculosis would have little impact on the general tuberculosis epidemic because MDR tuberculosis would always account for less than 10% of all tuberculosis (Fig. 2, Fig. 3, Fig. 4 and Table 1). In scenario 5, the combination of system change, new treatment regimen and new technology for drug-susceptible tuberculosis and optimized MDR tuberculosis care would yield the greatest reductions in tuberculosis incidence and mortality of 18.2% (95% CrI: 15.5 to 22.5%;) and 27.9% (95% CrI: 22.2 to 36.0%), respectively, in the decade beginning in 2015 (Table 1).

### Multidrug-resistant tuberculosis

**Fig. 5:**
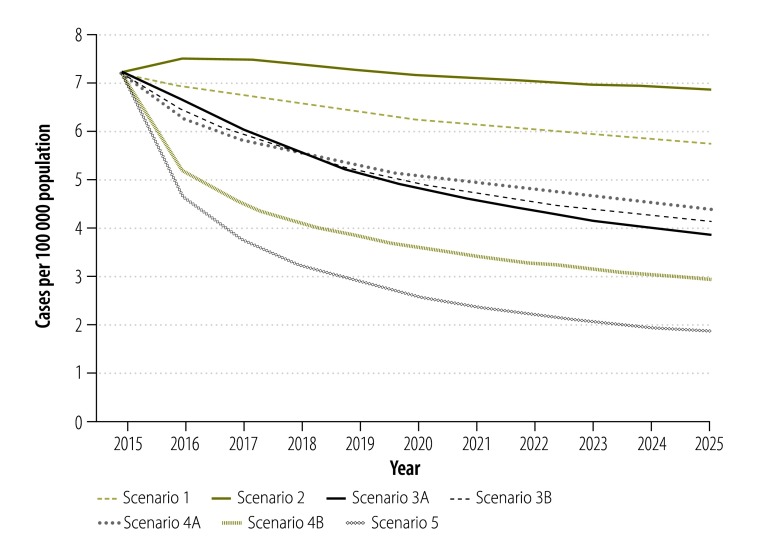
Projected impact of different scenarios of tuberculosis control on the prevalence of multidrug-resistant tuberculosis in the general population, China, 2015–2025

Note: The scenarios are as follows: 1: status quo; 2: shift from CCDC to hospital; 3A: all treated in CCDC; 3B: improved treatment of DS-TB; 4A: current best MDR-TB program; 4B: optimized MDR-TB program; 5: combined intervention.

**Fig. 6:**
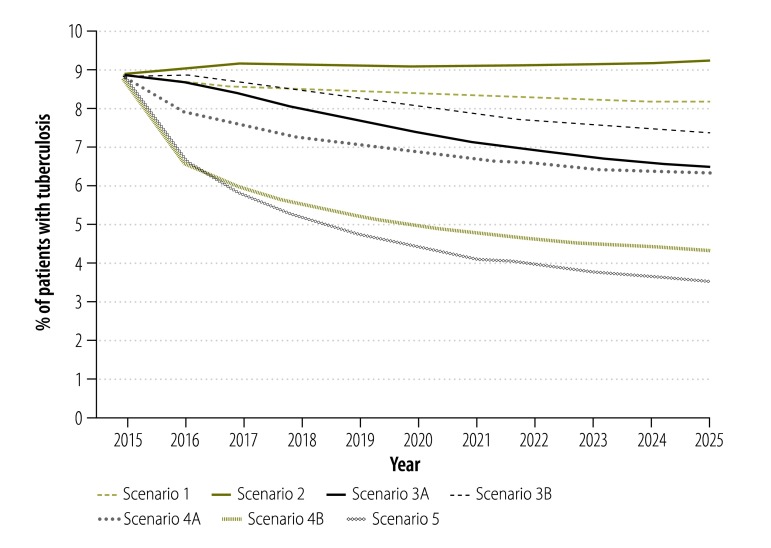
Projected impact of different scenarios of tuberculosis control on multidrug resistance among patients with tuberculosis, China, 2015–2025

Note: The scenarios are as follows: 1: status quo; 2: shift from CCDC to hospital; 3A: all treated in CCDC; 3B: improved treatment of DS-TB; 4A: current best MDR-TB program; 4B: optimized MDR-TB program; 5: combined intervention.

Under scenario 1, the absolute prevalence of MDR tuberculosis in the general population would be expected to decline over time – e.g. by 20.3% (95% CrI: 5.8 to 27.8%) by 2025 (Fig. 5). This decline would be mainly driven by the overall general decline in tuberculosis prevalence (Fig. 2) since the proportion of MDR tuberculosis would remain largely unchanged (Fig. 6). Improvement in the treatment outcomes for drug-susceptible tuberculosis and provision of diagnosis and treatment for MDR tuberculosis would further decrease the prevalence of MDR tuberculosis in the general population. In Scenario 5, the combination of system change, new treatment regimen and new technology would bring the greatest reduction in MDR tuberculosis prevalence in the general population of any of the modelled scenarios: a 74.6% (95% CrI: 62.6 to 80.8%) reduction between 2015 and 2025.

CCDC: Chinese Center for Disease Control and Prevention; DS-TB: drug-susceptible tuberculosis; MDR-TB: multidrug-resistant tuberculosis.

CCDC: Chinese Center for Disease Control and Prevention; DS-TB: drug-susceptible tuberculosis; MDR-TB: multidrug-resistant tuberculosis.

## Policy implications

### Post-2015 targets

The key finding from this analysis is that it is probably not possible for China to achieve the current global targets set for tuberculosis control.^4^ By 2010, however, China had already achieved the older global targets – of halving the prevalence and mortality of tuberculosis between 1990 and 2015.^1^^,^^18^ This achievement was mostly driven by shifting the treatment of tuberculosis from hospitals to the Chinese CDC public health centres.^1^ Our analysis suggests that the pace of reduction in tuberculosis incidence and mortality will be much slower in the future.

In the most optimistic of our scenarios (scenario 5) the incidence and mortality of tuberculosis would only be reduced by about 18% and 28%, respectively, between 2015 and 2025. This indicates that, with passive surveillance, current targets will not be reached by 2025 even if all the changes and interventions considered in our analysis were implemented. Active and enhanced surveillance might further accelerate the overall declines seen in tuberculosis, although the individual and community-level benefits are not clear.^19^

Looking beyond 2025, the global tuberculosis targets for 2035 – a 90% reduction in incidence and 95% reduction in mortality compared with the values seen in 2015 – appear even less attainable than the current, post-2015 targets. A new vaccine and/or prophylactic treatment to reduce the risk of the development of active disease developing among people with latent infection may well be needed to reach the 2035 targets.^4^^,^^20^

### Control of multidrug-resistant tuberculosis

The results of the 2007 National Survey of Tuberculosis Drug Resistance in China^3^ indicated a high proportion of MDR tuberculosis. There is no evidence from the 2010 survey of tuberculosis prevalence^1^ or the opinions of national tuberculosis experts to indicate that this proportion has since decreased. While attempting to integrate all of the available sources of relevant information, our analysis indicates that, under the status quo, this proportion will not change much by 2025 – although the absolute prevalence of MDR tuberculosis in the general population will decline as the overall prevalence of tuberculosis continues to fall. Further reductions in the prevalence of MDR tuberculosis in the general population might be achieved by improving treatment of drug-susceptible tuberculosis – thereby reducing the prevalence of tuberculosis and preventing the emergence of acquired resistance – or by providing MDR tuberculosis diagnosis and treatment and thereby reducing the transmission of MDR tuberculosis. Our analysis indicates that either of these interventions could accelerate the reduction of MDR tuberculosis prevalence in China and that the combination of both interventions would achieve the greatest impact: an estimated decline of 75% over the decade beginning in 2015.

### Model limitations

It is important to acknowledge the limitations of the modelling when interpreting the projected results. As with other modelling studies, the results of this analysis are sensitive to the parameter values and model structure. For example, we could not identify reliable information, from China, on the treatment outcomes of MDR tuberculosis using the first-line regimen or tuberculosis treated in the hospital system. For these variables, we used estimates of the national experts after they had reviewed the limited relevant data. Modelling provides an approach to make such estimates and assumptions explicit and to explore, using uncertainty analysis, the influence of assumptions and parameter uncertainty on the major findings. In our uncertainty analysis, we found that our qualitative conclusions were not affected by the uncertainty in most parameter values (available from the corresponding author).

### Impact on policy

China’s National Health and Family Planning Commission – formerly the Ministry of Health – is in the process of setting new, national, post-2015 tuberculosis-control targets and determining the next five-year plan for tuberculosis control, which will cover the period 2016 to 2020. The Chinese CDC is the main technical agency supporting this work. Our modelling analysis has provided the Chinese CDC with several important inputs for this process of policy development. First, it is clear that China cannot simply adopt the global post-2015 tuberculosis-control targets. The tuberculosis incidence and mortality targets for China will have to be more modest, partly because China has already greatly reduced its tuberculosis burden since 1990. Second, our analysis indicates that it should be possible to achieve a substantial reduction in the epidemic of MDR tuberculosis by improving the treatment of both drug-susceptible and MDR tuberculosis. Finally, our analysis indicates the importance of ensuring the high quality of tuberculosis treatment as patient care shifts from the Chinese CDC system to the hospital system.

## Conclusion

With new government policies, treatment of tuberculosis patients is being shifted from the Chinese CDC to designated hospitals. There is a risk that the treatment outcome in these designated hospitals will not achieve the level observed in the Chinese CDC system.^5^ If this is the case, the shift will have a negative impact on tuberculosis control. Our result highlights the importance of ensuring high treatment quality as the new policy to shift patients is implemented.

In the planning of post-2015 tuberculosis control in China, a rational process of policy development that is tailored to the country-specific context will be important. In this process, modelling could be used to integrate the different sources of relevant information and take account of any data uncertainty. A team of policy and field experts and modellers can facilitate the translation of modelling results into policy and practice. The approach that we followed may be useful in other settings and for other diseases.
